# The *Metallophosphoesterase-Domain-Containing Protein 2* (*MPPED2*) Gene Acts as Tumor Suppressor in Breast Cancer

**DOI:** 10.3390/cancers11060797

**Published:** 2019-06-08

**Authors:** Simona Pellecchia, Romina Sepe, Antonella Federico, Mariella Cuomo, Sara Carmela Credendino, Pasquale Pisapia, Claudio Bellevicine, Pedro Nicolau-Neto, Mariana Severo Ramundo, Elvira Crescenzi, Gabriella De Vita, Luigi Maria Terracciano, Lorenzo Chiariotti, Alfredo Fusco, Pierlorenzo Pallante

**Affiliations:** 1 Institute for Experimental Endocrinology and Oncology (IEOS) “G. Salvatore”, National Research Council (CNR), Via Sergio Pansini 5, 80131 Naples, Italy; simona.pellecchia@gmail.com (S.P.); romina.sepe@unina.it (R.S.); anfederi@unina.it (A.F.); cuomomariella.91@gmail.com (M.C.); e.crescenzi@ieos.cnr.it (E.C.); chiariot@me.com (L.C.); 2 Department of Molecular Medicine and Medical Biotechnology (DMMBM), University of Naples “Federico II”, Via Sergio Pansini 5, 80131 Naples, Italy; credendinosara@gmail.com (S.C.C.); gdevita@unina.it (G.D.V.); 3 Department of Public Health, University of Naples “Federico II”, Via Sergio Pansini 5, 80131 Naples, Italy; pasqualepisapia89@gmail.com (P.P.); claudiobellevicine@gmail.com (C.B.); 4 Instituto Nacional de Cancer, 37908, Laboratorio de Carcinogênese Molecular, Rua Andre Cavalcanti 37, Centro, Rio de Janeiro 20231-050, Brazil; pedronicolau.n@gmail.com (P.N.-N.); marianasevero@gmail.com (M.S.R.); 5 Institute of Pathology, Molecular Pathology Division, University of Basel, Schönbeinstrasse 40, 4031 Basel, Switzerland; luigi.terracciano@usb.ch

**Keywords:** MPPED2, MPPED2-AS1, methylation, breast cancer, long non-coding RNA, tumor suppressor

## Abstract

*Background*: We have recently reported the downregulation of the *Metallophosphoesterase-domain-containing protein 2 (MPPED2)* gene and its cognate long non-coding RNA, *MPPED2-AS1*, in papillary thyroid carcinomas. Functional studies supported a tumor suppressor role of both these genes in thyroid carcinogenesis. We then decided to investigate their role in breast carcinogenesis. *Methods*: In order to verify *MPPED2* expression, 45 human breast carcinoma samples have been investigated by quantitative real-time polymerase chain reaction (qRT-PCR). Then, *MPPED2* has been transfected in several human breast carcinoma cell lines, analyzing its role in cell proliferation, migration and invasion. To study the regulation of *MPPED2* expression the methylation of its promoter was investigated by targeted bisulfite sequencing. *Results*: *MPPED2* expression was decreased in breast cancer samples, and this was confirmed by the analysis of data available in The Cancer Genome Atlas (TCGA). Interestingly, the hypermethylation of *MPPED2* promoter likely accounted for its downregulation in breast cancer. Additionally, *MPPED2-AS1* was also found downregulated in breast cancer tissues and, intriguingly, its expression decreased the hypermethylation of the *MPPED2* promoter by inhibiting DNA methyltransferase 1 (DNMT1). Furthermore, the restoration of *MPPED2* expression reduced cell proliferation, migration and invasion capability of breast carcinoma cell lines. *Conclusion*: Taken together, these results propose *MPPED2* downregulation as a critical event in breast carcinogenesis.

## 1. Introduction

The *Metallophosphoesterase-domain-containing protein 2 (MPPED2)* belongs to the Class III of cyclic nucleotide phosphodiesterases (PDEs) and represents the first evidence of Class III-PDE identified in mammals [1]. This family of enzymes cleaves 3′,5′-cyclic phosphate nucleosides into 5′-phosphate nucleosides, regulating the cellular abundance of these cyclic second messengers and their degradation rates [2]. It has also been reported that MPPED2 protein exhibits low phosphodiesteric activity against cyclic adenosine 3′,5′-monophosphate (cAMP) and cyclic guanosine 3′,5′-monophosphate (cGMP), due to an aminoacidic replacement in the highly conserved catalytic site at position 252, where a glycine residue replaces a histydine [3]. This unique substitution allows the active site of MPPED2 to retain AMP or GMP with strong affinity, leading the protein to have low catalytic efficiency [4].

The human *MPPED2* gene is located between *FSHB* and *PAX6* genes on 11p13 chromosomal region, whose deletion has been found associated with WAGR syndrome, a pathological condition characterized by the presence of Wilms tumor, aniridia, genitourinary anomalies and mental retardation [5]. Interestingly, MPPED2 is highly conserved throughout the evolution with orthologs going from worms to humans supporting the critical role of this gene in biological functions [6]. Furthermore, it has been reported that *MPPED2* mRNA is highly expressed in foetal brain, suggesting its involvement in the development of the nervous system [7].

Although it has been reported that several PDE members are upregulated in cancer and have oncogenic activity [8,9], *MPPED2* was found to be downregulated in several cancer types, such as neuroblastoma [10], cervical cancer [11] and oral squamous carcinomas [12]. Recently, our group has reported that *MPPED2* is downregulated in benign and malignant thyroid neoplasia, and that its restoration in thyroid carcinoma cell lines reduced cell proliferation and migration [13]. Moreover, a positive correlation was also reported between *MPPED2* and *MPPED2 antisense RNA1 (MPPED2-AS1*, previously indicated as *RP5-1024C24.1*), a long non-coding RNA (lncRNA) gene located on the same chromosomal region in antisense position with respect to the *MPPED2* gene.

Therefore, in order to verify whether *MPPED2* downregulation was a more general event in cancer, we evaluated *MPPED2* expression in breast cancer (BC), aiming also to identify new players in breast carcinogenesis that might represent useful diagnostic markers.

BC is the most common female cancer worldwide and remains a leading cause of death for cancer among women both in developed and developing regions with an estimated 2.1 million of new cancer cases diagnosed in 2018 [14]. BC is subdivided into several subtypes defined by the presence/absence of estrogen receptor (ER), progesterone receptor (PR) and human epidermal growth factor receptor 2 (HER2), key genes that play crucial roles in mammary oncogenesis [15,16]. The molecular BC subtypes are classified in luminal A (Lum A: ER+ and/or PR+, HER2–), luminal B (Lum B: ER+ and/or PR+, HER2+), HER2 enriched (HER2: ER–, PR–, HER2+) and triple-negative breast cancer (TNBC: ER−, PR−, HER2−) subtypes. TNBC subtype is additional subdivided in basal-like (enriched with basal marker) and claudin-low (overexpressing genes correlated with aggressive behavior) subtypes [17,18,19]. However, despite considerable improvement in screening, diagnosis and therapy, the molecular bases of BC development remain unknown.

In the present study we show that *MPPED2* expression was decreased in BC samples, likely due to its hypermethylated promoter, reflecting data available on The Cancer Genome Atlas (TCGA). Intriguingly, the downregulation of *MPPED2-AS1* was also observed in BC. In this context, we report that *MPPED2-AS1* interacted with DNA methyltransferase 1 (DNMT1), thus modulating the methylation of *MPPED2* promoter. To further characterize *MPPED2* role we performed functional assays in MDA-MB-231 (claudin-low), SKBR3 (HER2) and MDA-MB-468 (basal-like) [17,18,19], being cell lines in which *MPPED2* showed the lowest expression levels with respect to the other cell lines analyzed (MCF7, Lum A and BT-474, Lum B). Additionally, MCF7 cells were also used to analyze the role of *MPPED2* in less aggressive BC cells. Functional assays clearly demonstrated that *MPPED2* overexpression reduced cell proliferation, migration and invasion capability, thus supporting a critical role of *MPPED2* downregulation in breast carcinogenesis where *MPPED2* would act as tumor suppressor gene.

## 2. Results

### 2.1. MPPED2 and MPPED2-AS1 Are Downregulated in Human Breast Carcinoma Tissues

**Figure 1:**
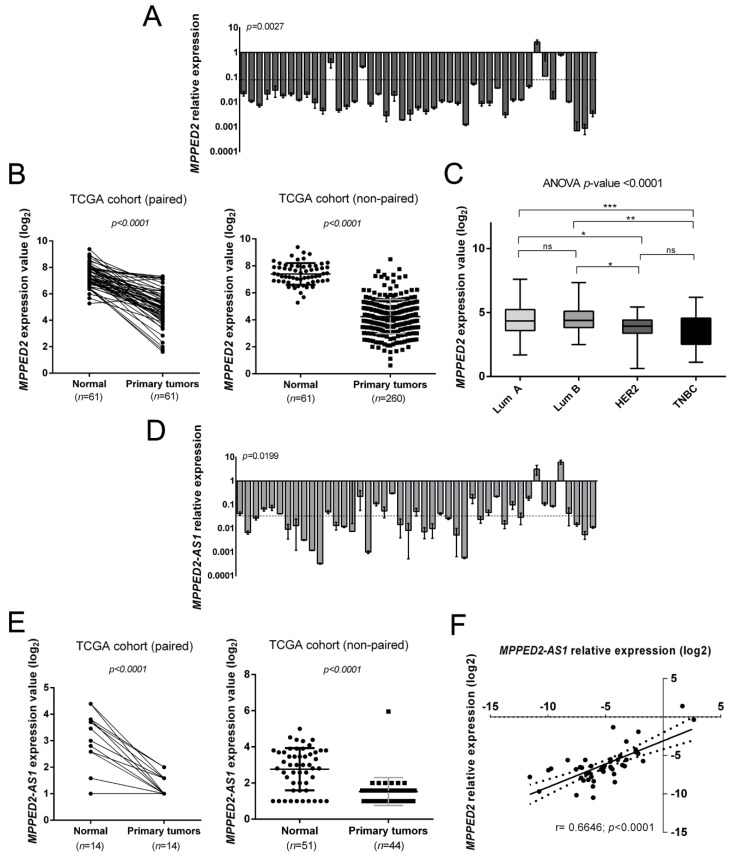
Expression of *MPPED2* and *MPPED2-AS1* in human breast carcinoma tissues. (**A**) *MPPED2* expression levels were evaluated by quantitative real-time polymerase chain reaction (qRT-PCR) in 45 breast carcinoma tissues. The dashed line represents the average relative expression value of the analyzed samples (*p* = 0.0027). Data are reported as 2^−ΔΔCt^ values ± standard deviation (SD), compared to the mean of breast normal tissues, set equal to 1. (**B**) *MPPED2* expression levels were evaluated in a dataset available in The Cancer Genome Atlas (TCGA). Paired (left panel) and non-paired (right panel) breast cancer samples were analyzed. *t*-test: ****, *p* < 0.0001 (primary tumors vs. normal tissues in both paired and non-paired samples). (**C**) *MPPED2* expression levels were evaluated in the molecular subtypes of breast carcinoma tissues (Lum A, Lum B, HER2 and TNBC) in the TCGA dataset. Box and whiskers: Min to max. One-way analysis of variance (ANOVA) test: ****, *p* < 0.0001. (**D**) *MPPED2-AS1* levels were evaluated in 45 breast carcinoma tissues by qRT-PCR. The dashed line represents the average relative expression value of the analyzed samples (*p* = 0.0199). Data are reported as 2^−ΔΔCt^ values ± SD, compared to the mean of breast normal tissues, set equal to 1. (**E**) *MPPED2-AS1* expression levels were evaluated in the TCGA dataset. Paired (left panel) and non-paired (right panel) breast cancer samples were evaluated and only samples in which *MPPED2-AS1* was detectable were considered for the analysis. *t*-test: ****, *p* < 0.0001 (primary tumors vs. normal tissues in both paired and non-paired samples). (**F**) Correlation scatter plot (Spearman’s Rank) between qRT-PCR levels of *MPPED2* and *MPPED2-AS1* analyzed in 45 breast carcinoma samples (r = 0.6646; ****, *p* < 0.0001).

We evaluated *MPPED2* mRNA levels in a panel of 45 human BC tissues by quantitative real-time polymerase chain reaction (qRT-PCR). The results, shown in Figure 1A, evidenced a drastic downregulation of *MPPED2* expression in 44 out of 45 human BC samples when compared to normal breast tissues, with an average relative expression value of 0.10 (**, *p* = 0.0027). Then, we also evaluated *MPPED2* expression in both paired and non-paired BC tissues in a dataset available in the TCGA database. Accordingly, *MPPED2* expression was significantly lower in 260 primary BC samples in comparison with normal breast tissues (****, *p* < 0.0001), as well as in 61 BC samples in comparison with matched breast normal tissues (****, *p* < 0.0001) (Figure 1B). In addition, *MPPED2* expression levels were also analyzed in the different BC molecular subtypes of the TCGA cohort (Lum A, Lum B, HER2 and TNBC): as reported in Figure 1C, *MPPED2* was found to be downregulated in all molecular subtypes (****, *p* < 0.0001), in particular in HER2 and in TNBC ones. Moreover, *MPPED2* reduction resulted even lower in ER- respect to ER+ BC samples (Supplementary Materials Figure S1C).

Subsequently, we assessed the *MPPED2-AS1* expression levels in the same set of BC samples by qRT-PCR. Interestingly, *MPPED2-AS1* levels were downregulated in 43 out of 45 analyzed samples, with an average relative expression value of 0.05 (*, *p* = 0.0199) (Figure 1D). Furthermore, we evaluated the *MPPED2-AS1* levels in the same TCGA cohort utilized for *MPPED2* and, we observed that *MPPED2-AS1* was downregulated in both paired (****, *p* < 0.0001) and non-paired (****, *p* < 0.0001) BC samples. Moreover, in a large number of samples *MPPED2-AS1* levels were not detectable at all (Supplementary Materials Figure S1A). However, considering only the samples in which *MPPED2-AS1* expression was detectable, we still found a significant downregulation in both paired (****, *p* < 0.0001) and non-paired (****, *p* < 0.0001) BC samples (Figure 1E). Besides, *MPPED2-AS1* expression was also analyzed in the BC molecular subtypes of the TCGA cohort, even though any significant difference was found among the groups (Supplementary Materials Figure S1B).

Then, we investigated whether a possible correlation between *MPPED2* and *MPPED2-AS1* expression occurred. As expected, a significant positive correlation (****, *p* < 0.0001) was found in BC samples analyzed by qRT-PCR (Figure 1F). These results suggest that the co-regulation of *MPPED2* and *MPPED2-AS1* genes also occurs in BC.

**Figure 2:**
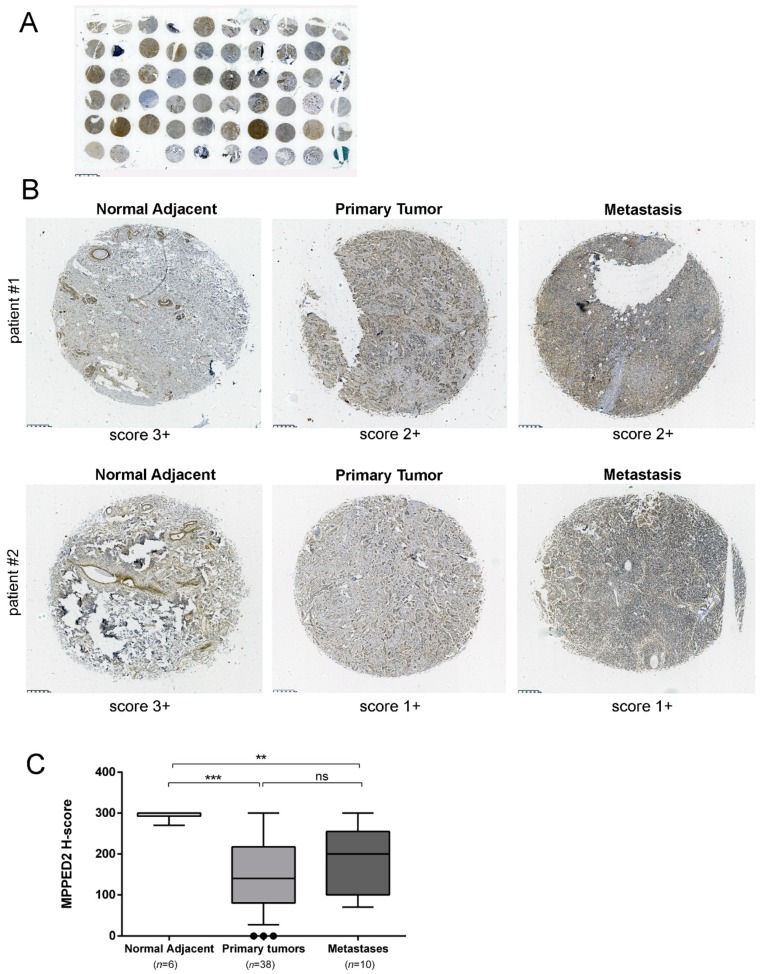
MPPED2 protein expression in human breast carcinoma tissues. (**A**) Gross view of immunohistochemical staining of a tissue microarray (TMA) slide. Scale bar, 2.5 mm. (**B**) Representative immunohistochemical staining of MPPED2 protein in normal adjacent tissue (left), breast primary tumor (center) and metastatic tissue (right) derived from two different patients (upper and lower panel), respectively. MPPED2 signal is strong in normal adjacent tissue (score 3+) and moderate (score 2+) or weak (score 1+) in primary tumor or metastatic tissues (magnification 200×). Scale bar, 250 µm. (**C**) Intensity of the staining and the percentage of positive cells were combined to obtained H-score. Box and whiskers: 10–90 percentile; Mann–Whitney test: ***, *p* < 0.001 (primary tumor vs. normal adjacent); **, *p* < 0.01 (metastasis vs. normal adjacent); ns, not significant (primary tumor vs. metastasis).

Finally, we evaluated MPPED2 protein expression in a tissue microarray (TMA) containing breast carcinoma tissues (*n* = 38), metastases (*n* = 10) and normal adjacent breast tissues (*n* = 6) by immunohistochemical (IHC) staining with a specific antibody against MPPED2. A gross view of IHC staining of TMA slide is shown in Figure 2A. The results of the IHC were summarized in Table 1 and two representative IHC stainings are shown in Figure 2B. The intensity of the signal corresponding to MPPED2 was reduced in matched breast carcinoma samples (center upper panel, score 2+; center lower panel, score 1+) and metastases (right upper panel, score 2+; right lower panel, score 1+) when compared to the normal adjacent tissues (left, upper and lower panel, score 3+). For each spot, we also evaluated the percentage of positive cells and this parameter was combined with the intensity score to calculate H-score (Materials and Methods). H-score analysis confirmed that MPPED2 expression was significantly reduced in tumor tissues (***, *p* < 0.001) and metastases (**, *p* < 0.01), compared with normal adjacent breast tissues (Figure 2C). No significant association was found between MPPED2 expression and tumor size, histological grade, lymph node, TNM stage and the status of ER, PR and HER2 (Supplementary Materials Table S1), while a significant one was found with age (Fisher’s exact test, *p* = 0.0002, Supplementary Materials Table S1).

### 2.2. Hypermethylation of MPPED2 Promoter Accounts for MPPED2 Downregulation in Breast Cancer

**Figure 3:**
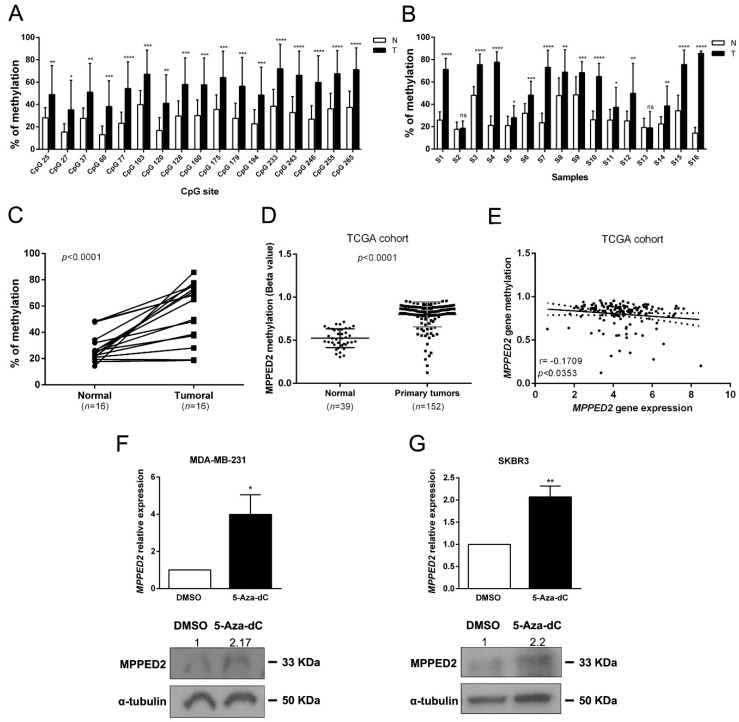
*MPPED2* methylation status in normal and tumoral samples. (**A**) Methylation levels of CpG sites in the *MPPED2* promoter. Each histogram represents the mean value of each CpG site in the whole cohort analyzed. White and black bars represent normal and breast cancer tissues, respectively. *t*-test: *, *p* < 0.05; **, *p* < 0.01; ***, *p* < 0.001; ****, *p* < 0.0001. (**B**) Methylation levels of the *MPPED2* promoter in each sample. Histograms represent the mean methylation value of all CpG sites in each sample. White and black bars represent normal and breast cancer tissues, respectively (S1–S16, different patient samples). *t*-test: *, *p* < 0.05; **, *p* < 0.01; ***, *p* < 0.001; ****, *p* < 0.0001; ns, not significant. (**C**) Percentage of methylation of each tumoral sample compared to matched normal sample. *t*-test: ****, *p* < 0.0001. (**D**) *MPPED2* methylation levels were evaluated in a dataset available at The Cancer Genome Atlas (TCGA) comprising 152 primary breast tumors and 39 normal breast samples. *t*-test: ****, *p* < 0.0001. (**E**) Correlation scatter plot (Spearman’s Rank) analysis between *MPPED2* methylation values and expression levels in the TCGA cohort (r = −0.1709; *p* < 0.0353). (**F**,**G**) *MPPED2* expression levels were evaluated by qRT-PCR (upper panel) in MDA-MB-231 and SKBR3 cell lines after 2 µM 5-Aza-dC or dimethyl sulfoxide (DMSO) treatment for 120 h. Data were reported as mean ± SD. *t*-test: *, *p* < 0.05 (5-Aza-dC vs. DMSO, MDA-MB-231); **, *p* < 0.01 (5-Aza-dC vs. DMSO, SKBR3). MPPED2 protein expression was also evaluated by Western blot (lower panel). Densitometric analysis of a representative experiment was performed by using ImageJ software. MPPED2 protein expression in 5-Aza-dC treatment was compared to DMSO treatment, set equal to 1. Original blots were shown in Supplementary Materials Figure S4.

In order to unveil the mechanisms leading to the reduced *MPPED2* expression in BC tissues we evaluated the methylation of a CpG island located upstream of the transcriptional start site (TSS) of the *MPPED2* gene. We carried out next generation sequencing on 16 genomic DNAs deriving from matched tumoral and normal breast tissues, after treatment with bisulfite. As shown in Figure 3A, 17 CpG sites were strongly hypermethylated in tumoral samples. Furthermore, 14 out of 16 tumoral samples (87.5%) analyzed showed a strong hypermethylation of the analyzed CpG sites when compared to the matched normal tissues (Figure 3B,C). Then, we evaluated the methylation of *MPPED2* promoter on the same TCGA BC cohort previously considered for *MPPED2* gene expression. Consistently, we found that *MPPED2* promoter was strongly methylated in 152 tumoral samples compared to 39 normal ones (Figure 3D). Intriguingly, from the TCGA analysis it was also clear that the suppression of *MPPED2* expression was more pronounced in tumors with CpG hypermethylation. In fact, as shown in Figure 3E, we observed a significant negative correlation between *MPPED2* expression and promoter methylation (r = −0.1709; *, *p* < 0.0353). Therefore, these results suggest that *MPPED2* expression might be mainly regulated through the methylation of its promoter. To validate this hypothesis, *MPPED2* expression levels were evaluated by qRT-PCR in a panel of BC-derived cell lines, including MDA-MB-231 and MDA-MB-468 (TNBC), SKBR3 (HER2), MCF7 (Lum A) and BT-474 (Lum B) (Supplementary Materials Figure S2). MDA-MB-231 and SKBR3 cells, in which *MPPED2* expression was extremely low (Supplementary Materials Figure S2), were treated for 120 h with 5-Aza-2′-deoxycytidine (5-Aza-dC) and levels of *MPPED2* mRNA were then evaluated. Consistent with our hypothesis, re-expression of *MPPED2* was obtained in MDA-MB-231 (*, *p* < 0.05) and SKBR3 (**, *p* < 0.01) cells after 5-Aza-dC treatment (Figure 3F,G), confirming that the downregulation of *MPPED2* expression in BC could be mainly due to the hypermethylation of *MPPED2* regulatory regions.

### 2.3. MPPED2-AS1 Modulates MPPED2 Expression through DNA Methylation

**Figure 4:**
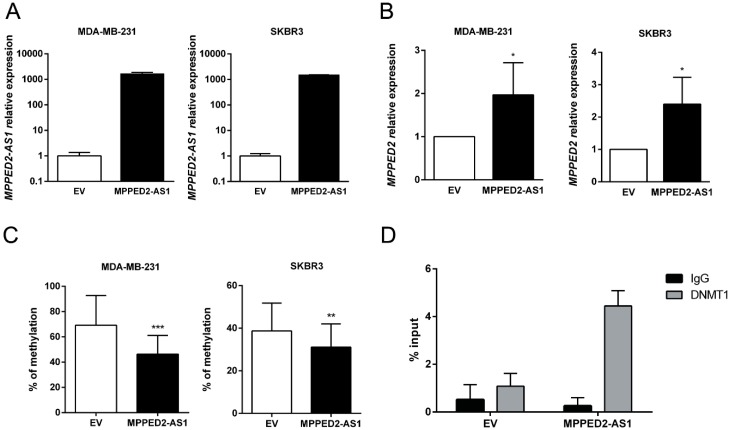
*MPPED2-AS1* interacts with DNMT1 decreasing *MPPED2* promoter methylation. (**A**) qRT-PCR analysis of *MPPED2-AS1* expression levels in MDA-MB-231 and SKBR3 cells transfected with *MPPED2-AS1* overexpressing vector or the empty vector (EV). Data are reported as 2^−ΔΔCt^ values ± SD, compared to the EV, set equal to 1. (**B**) qRT-PCR analysis of *MPPED2* mRNA levels in MDA-MB-231 and SKBR3 cells transfected with *MPPED2-AS1* overexpressing vector or the empty vector (EV). Data are reported as 2^−ΔΔCt^ value ± SD, compared to EV, set equal to 1. *t*-test: *, *p* < 0.05 (MDA-MB-231 and SKBR3). (**C**) *MPPED2* promoter methylation levels were evaluated in MDA-MB-231 and SKBR3 cells transfected with *MPPED2-AS1* overexpressing vector or the empty vector (EV). *t*-test: ***, *p* < 0.001 (MDA-MB-231); **, *p* < 0.01 (SKBR3). (**D**) RNA immunoprecipitation was performed on extracts obtained from SKBR3 cells transfected with *MPPED2-AS1* overexpressing vector or the empty vector (EV), using an anti-DNMT1 antibody or a pre-immune (IgG) serum, as control. Immunoprecipitated *MPPED2-AS1* RNA was quantified by qRT-PCR. RNA levels were reported as percentage of input and were calculated with the 2^−ΔCt^ formula.

Since *MPPED2* and *MPPED2-AS1* expression levels were downregulated and positively correlated in BC samples, we hypothesized that *MPPED2* expression can be regulated by *MPPED2-AS1* through an epigenetic mechanism. To this end, we restored *MPPED2-AS1* expression in both MDA-MB-231 and SKBR3 cells (Figure 4A), testing them for *MPPED2* expression by qRT-PCR. As shown in Figure 4B, a significant increase of *MPPED2* mRNA levels were observed after *MPPED2-AS1* overexpression, confirming that the lncRNA plays an important role in *MPPED2* regulation. Then, we investigated the methylation of the CpG island located upstream of the *MPPED2* TSS. The data showed that the overexpression of *MPPED2-AS1* leads to a reduction of *MPPED2* promoter methylation in both BC cell lines, suggesting that this lncRNA may control *MPPED2* methylation (Figure 4C).

Since DNMT1 is a crucial factor for mammary carcinogenesis, being involved in several aspects of tumorigenesis [20,21,22], we verified whether *MPPED2-AS1* acts via DNMT1. In order to analyze the physical interaction between *MPPED2-AS1* and DNMT1, RNA immunoprecipitation (RIP) assay was performed by using a specific anti-DNMT1 antibody in SKBR3 cells. Consistently, *MPPED2-AS1* was enriched after DNMT1 immunoprecipitation, compared to the control group (IgG), confirming that this lncRNA physically interacts with DNMT1 (Figure 4D). Taken together, these results show that the interaction between *MPPED2-AS1* and DNMT1 may prevent the methylation of CpG islands in *MPPED2* promoter region, thus avoiding its reduction.

### 2.4. Restoration of MPPED2 Expression Reduces Cell Growth Rate and Colony Formation in Human Breast Cancer Cell Lines

**Figure 5:**
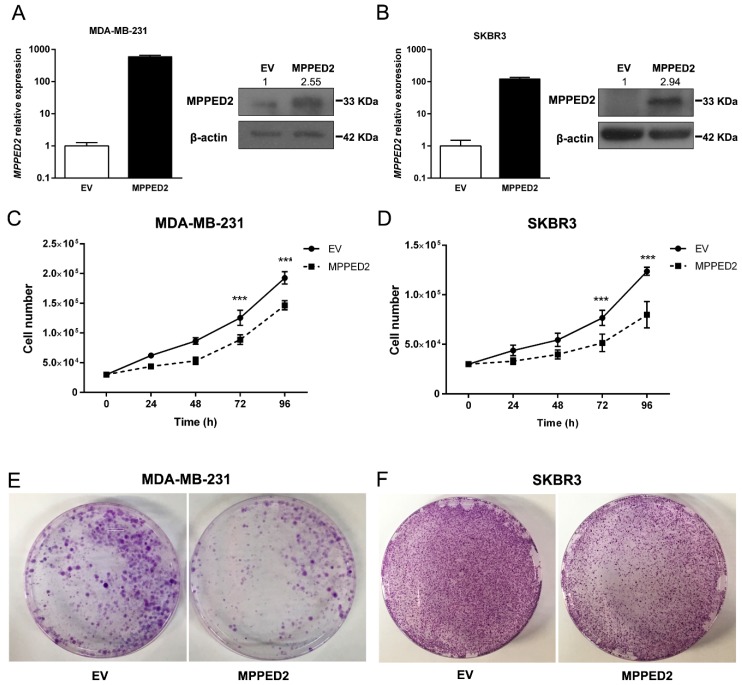
*MPPED2* reduces cell proliferation of breast carcinoma cell lines. (**A**,**B**) qRT-PCR (left panel) performed in MDA-MB-231 and SKBR3 cells stably expressing *MPPED2* or carrying the corresponding empty vector (EV). Data are reported as 2^−ΔΔCt^ values ± SD, compared to the EV, set equal to 1. Western bot (right panel) analysis confirming the expression of MPPED2. β-actin was used to normalize the amount of loaded protein (left panel). Densitometric analysis was performed by using ImageJ software to evaluate MPPED2 protein expression compared to EV, set equal to 1. Original blots were shown in Supplementary Materials Figure S5. (**C**,**D**) Cell growth analysis of MDA-MB-231 and SKBR3 cells stably expressing *MPPED2* or carrying the corresponding empty vector (EV). Cell number was evaluated at 24 h, 48 h, 72 h and 96h after seeding. Values were obtained from three independent experiments performed in duplicate. Data are reported as mean ± SD. 2-way ANOVA test (Bonferroni post-test: *MPPED2* vs. EV, 96 h and 72 h, ***, *p* < 0.001). (**E**,**F**) Representative colony assays performed in MDA-MB-231 and SKBR3 cells transiently transfected with *MPPED2* or the corresponding empty vector (EV). Cell were stained with crystal violet after 3 weeks of selection with hygromycin.

To define the role of *MPPED2* downregulation in breast carcinogenesis we investigated the effects of *MPPED2* overexpression in cell proliferation and cell-cycle regulation. To perform growth curve analyses and colony formation assays, we restored *MPPED2* expression in MDA-MB-231 and SKBR3 cells, confirming its overexpression by qRT-PCR and Western blot (Figure 5A,B). As shown in Figure 5C,D, *MPPED2* stably transfected cells grew significantly more slowly than the control cells transfected with the empty vector (EV). Consistently, colony assay experiments confirmed that *MPPED2* overexpression inhibited the formation of colonies (Figure 5E,F).

Moreover, we performed growth curve assay also in MDA-MB-468 and MCF7 cells transiently transfected with *MPPED2* to determine whether MPPED2 could affect also less aggressive BC cell lines. After confirming by qRT-PCR and Western blot analyses the MPPED2 overexpression in transiently transfected cells (Supplementary Materials Figure S3A,B), we observed that the restoration of *MPPED2* was able to inhibit the growth in both cell lines when compared to the control cells (Supplementary Materials Figure S3C), leading us to consider that its tumor suppressor activity is not restricted to the more aggressive behavior of BC cells.

**Figure 6:**
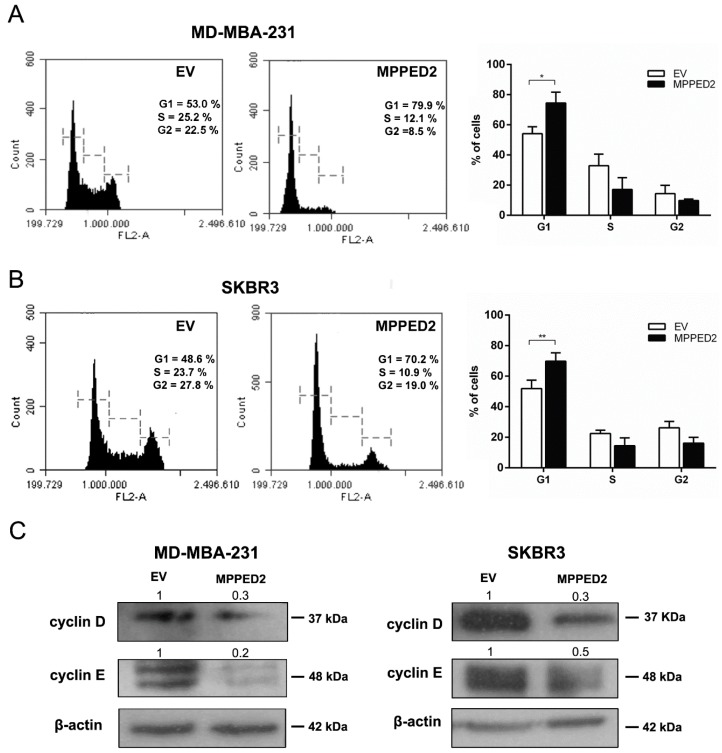
*MPPED2* inhibits cell cycle progression of breast carcinoma cell lines. (**A**,**B**) Representative experiments of cell cycle analysis performed in MDA-MB-231 and SKBR3 cells stably transfected with *MPPED2* or the corresponding empty vector (EV) are shown in left panel. Values shown in the right panel were obtained from three independent experiments. *t*-test: *, *p* = 0.0286 (*MPPED2* vs. EV, MDA-MB-231 G1 phase); **, *p* = 0.0079 (*MPPED2* vs. EV, SKBR3 G1 phase). (**C**) Western blot analysis of cyclin D and cyclin E expression in MD-MBA-231 (left panel) and SKBR3 (right panel) stably expressing *MPPED2* or carrying the corresponding empty vector (EV). β-actin was used to normalize the amount of loaded protein. Densitometric analysis was performed by using ImageJ software to analyse protein expression compared to the EV, set equal to 1. Original blots were shown in Supplementary Materials Figure S6.

Finally, flow cytometry analysis was performed to investigate whether *MPPED2* overexpression influenced cell cycle. As shown in Figure 6A,B (right panel), *MPPED2* overexpression induced a significant increase in G1 phase of MDA-MB-231 (74.4% vs. 54.15%; *, *p* = 0.0286, *MPPED2* vs. control) and SKBR3 (69.82% vs. 51.88%; **, *p* = 0.0079, *MPPED2* vs. control) cell number. The profile of a representative experiment is shown in Figure 6A,B (left panel). Furthermore, in accordance with flow cytometry data, reduced cyclin D1 and cyclin E protein levels were observed in both MDA-MB-231 and SKBR3 cells overexpressing *MPPED2* compared with the control cell transfected with the empty vector (Figure 6C). Therefore, these results confirmed that MPPED2 was able to negatively regulate BC cell proliferation through the accumulation of cells in the G1 phase of the cell cycle.

### 2.5. MPPED2 Expression Suppresses Cell Migration and Invasion

**Figure 7:**
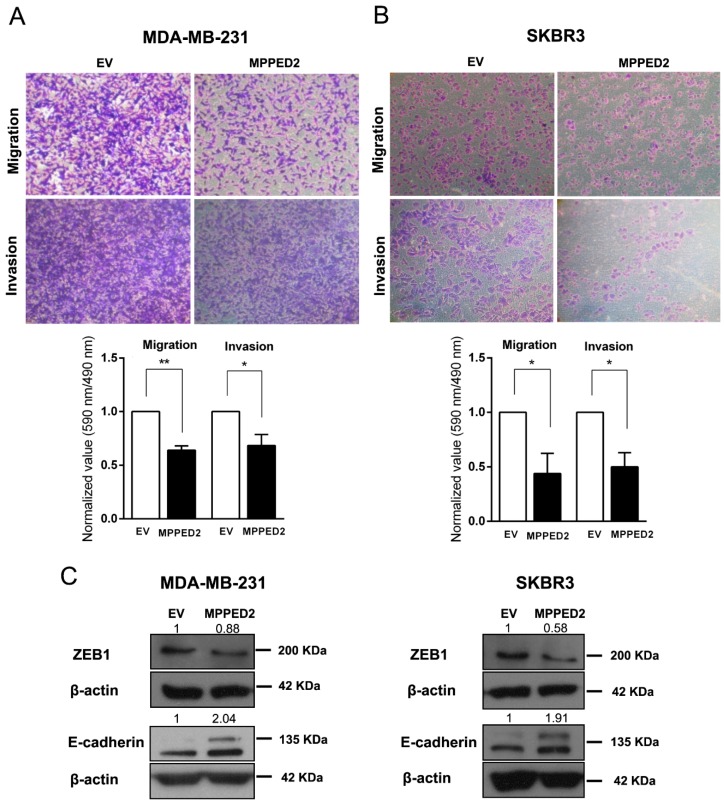
*MPPED2* reduces cell migration and invasion of breast carcinoma cell lines. (A,B) Representative images of migration and invasion assays performed in MDA-MB-231 and SKBR3 cells stably transfected with *MPPED2* or the corresponding empty vector (EV) (upper panel). Magnification 40×. Migrated or invaded cells were fixed with crystal violet solution. After elution from transwell, it was quantified by reading its absorbance at 590 nm. Crystal violet absorbance values were divided by corresponded cell titer values (Materials and Methods) and were plotted in *y*-axis. Data obtained from three independent experiments carried out in MDA-MB-231 and SKBR3 cells are shown in the lower panel. Values are reported as mean value ± SD, compared to the EV, set equal to 1. *t*-test: *, *p* < 0.05, ** *p* < 0.01. (C) Western blot analysis of ZEB1 and E-cadherin in MD-MBA-231 (left panel) and SKBR3 (right panel) cells stably expressing *MPPED2* or carrying the corresponding empty vector (EV). β-actin was used to normalize the amount of loaded protein. Densitometric analysis was performed by using ImageJ software to evaluate protein expression compared to EV, set equal to 1. Original blots were shown in Supplementary Materials Figure S7.

Next, to determine whether *MPPED2* expression may affect cell migration and invasion, we performed transwell and Matrigel invasion assays on MDA-MB-231 and SKBR3 cells stably transfected with *MPPED2*. Indeed, *MPPED2* overexpression reduced cell migration and invasion ability of about 40% (**, *p* < 0.01) and 32% (*, *p* < 0.05), respectively in MDA-MB-231 (Figure 7A). These data were also confirmed in SKBR3 overexpressing *MPPED2*, where migration and invasion were reduced of about 60% (*, *p* < 0.05) and 50% (*, *p* < 0.05), respectively (Figure 7B). Reduction of migration ability was also confirmed in MDA-MB-468 and MCF7 cells (Figure S3D).

Finally, to explain the mechanism by which *MPPED2* affects migration and invasion, we performed Western blot analyses of ZEB1 and E-cadherin proteins, two important epithelial–mesenchymal transition (EMT) markers [23,24,25]. Interestingly, we found that the restoration of *MPPED2* in MDA-MB-231 and SKBR3 cells led to the reduction of ZEB1 and to the increase of E-cadherin protein levels (Figure 7C). Thus, we can speculate that *MPPED2* expression reduces BC cell migration and invasion *in vitro* through the modulation of these canonical EMT markers, further supporting its anti-oncogenic role played in breast carcinogenesis.

## 3. Discussion.

The aim of this study was to investigate whether the tumor suppressor role of MPPED2 described by our group in thyroid neoplasia could be extended to BC. The results reported here clearly indicate that the *MPPED2* gene plays an anti-oncogenic role in human BC. Indeed, we demonstrated that *MPPED2* was strongly downregulated in almost all BC samples analyzed with respect to the normal breast tissues. This result was even confirmed on a larger number of cases through the evaluation of data available in the TCGA database. *MPPED2* expression did not correlate with any clinico-pathological feature of BC patients and this excludes the possibility of using MPPED2 detection as prognostic marker. However, its detection through qRT-PCR and IHC might represent a new tool for the diagnosis of breast neoplasia.

Epigenetic alterations, including DNA methylation, represent one of the greatest molecular changes that occur in several malignancies, including BC [26]. In fact, CpG islands located upstream of the promoter regions of tumor suppressor genes are generally hypermethylated in tumoral cells. This is frequently observed in several human cancers including BC, and it is responsible for the transcriptional silencing of these genes. Therefore, this occurrence prompted the scientific community to considerer the hypermethylation of CpG islands as target for clinical strategies [27,28]. Considering this, we have investigated the possible mechanism responsible for the downregulation of *MPPED2* expression by analyzing the methylation of its promoter. Hypermethylation of the promoter was detected in 14 out of 16 BC cases analyzed, and this finding was also confirmed through analysis of data available in TCGA database. Consistently, the treatment of BC cells with the demethylating agent 5-Aza-dC restored *MPPED2* expression, supporting the hypothesis that *MPPED2* hypermethylation accounted for its downregulation in BC. Interestingly, we also reported that the lncRNA *MPPED2-AS1* expression was downregulated and positively correlated with *MPPED2* in BC, as already demonstrated by our group in papillary thyroid carcinomas. Here, we demonstrate that the overexpression of *MPPED2-AS1* was able to reduce the methylation levels of *MPPED2* promoter in MDA-MB-231 and SKBR3 cells. Additionally, we also demonstrate that *MPPED2-AS1* was able to bind DNMT1, inhibiting its activity. Then, the *MPPED2-AS1* reduced expression would lead to an increased methyltransferase activity, with a consequent hypermethylation of the *MPPED2* promoter. However, we cannot exclude that other epigenetic mechanisms might be involved in the regulation of the *MPPED2* gene expression.

Subsequently, functional studies demonstrated that the restoration of *MPPED2* in BC cells, in which its expression was very low, significantly reduced cell growth rate arresting the cells in the G1 phase, with a reduced cyclin D and cyclin E expression. Moreover, *MPPED2* expression negatively affected BC cell migration and invasion, supporting the anti-oncogenic role of MPPED2 in breast carcinogenesis. Interestingly, to determine whether *MPPED2* could impact on migration and invasion through the modulation of EMT proteins, we found that its restoration was able to reduce ZEB1 and to increase E-cadherin protein levels, thus suggesting that *MPPED2* could exert its action through this cell signaling pathway [23,24,25]. However, the mechanism by which MPPED2 exerts its tumor suppressor activity requires further studies.

Previous studies have evidenced *MPPED2* downregulation also in neuroblastoma [10], cervical cancer [11], oral squamous carcinoma [12] and thyroid cancer [13], indicating that its tumor suppressor activity is not confined to few neoplastic histotypes. Noteworthy, preliminary results obtained by our research group have reported analogous results also in human glioblastomas and colorectal carcinomas (data not shown) (S.P. (DMMBM, Naples, Italy); P.P. (IEOS-CNR, Naples, Italy); A.F. (DMMBM, Naples, Italy). Personal communication, 2019). However, we could exclude *MPPED2* downregulation as a feature of all human malignancies, since liver, lung and prostate carcinomas did not display any decrease of *MPPED2* expression, as evaluated on TCGA dataset. Finally, the physiological role of MPPED2 during normal development and in adult tissues is still far from being defined: the generation of knock out mice for the *MPPED2* gene would surely give more information about the physiological role of *MPPED2* and may even validate its tumor suppressor role.

## 4. Materials and Methods

### 4.1. Human Breast Tissue Samples

Normal and neoplastic human breast tissues were obtained from surgical specimens and immediately frozen in liquid nitrogen. Breast samples were kept frozen until required for nucleic acid extraction. Breast tissue samples were collected at the Institute of Pathology, University of Basel, Switzerland. The study was conducted under the approval of the local ethical committee (#78-09). Informed consent was obtained from all patients.

### 4.2. Cell Culture and Transfection

Human breast cancer cell lines MDA-MB-231, SKBR3, MDA-MB-468 and MCF7 were cultured in Dulbecco’s Modified Eagle Medium (DMEM) (Sigma-Aldrich, St. Louis, MO, USA) supplemented with 10% foetal bovine serum (FBS) (Euroclone, Milan, Italy), 1% L-glutamine, 1% penicillin/streptomycin (Sigma-Aldrich). Cells were kept at 37 °C under 5% CO_2_ atmosphere. Lipofectamine 2000 (Life Technologies, Grand Island, NY, USA) reagent was used to transfect the cells according to the manufacturer’s instructions. The stably MDA-MB-231 and SKBR3 transfected cells were selected in a medium containing 600 µg/mL and 100 µg/mL of hygromycin (Sigma-Aldrich), respectively.

### 4.3. Plasmids

The expression vector encoding human *MPPED2* gene was generated by cloning cDNA sequence in the pcDNA3.1/Hygro (+) vector (Life Technologies) using HindIII and NotI restriction sites. After cloning, the plasmid was subjected to sequencing (Eurofins Genomics, Vimodrone, Italy) and MPPED2 expression was validated by qRT-PCR and Western blot analyses. The expression vector encoding human *MPPED2-AS1* was obtained by cloning the lncRNA sequence in the pCMV6-AC-GFP vector (Origene Technologies, Rockville, MD, USA) using the HindIII and XhoI restriction sites [13].

### 4.4. The Cancer Genome Atlas (TCGA) Database

*MPPED2* expression data for BC samples were obtained from The Cancer Genome Atlas (TCGA) by using web-based software Wanderer [29]. From the whole cohort, a total of 260 primary BC and 61 normal breast surrounding tissues were considered for this study. Patients were classified based on the BC subtype (Lum A, *n* = 108; Lum B, *n* = 65; HER2, *n* = 35; TNBC, *n* = 52). For the methylation analysis *n* = 39 normal tissues and *n* = 152 primary tumors were examined. *MPPED2-AS1* expression data for BC samples were obtained from TCGA repository (https://portal.gdc.cancer.gov/repository), using the same BC cohort utilized for the evaluation of *MPPED2* expression levels. A total of 250 primary BC and 61 normal breast adjacent tissues were considered for this study. Patients were classified based on the BC subtype (Lum A, *n* = 103; Lum B, *n* = 62; HER2, *n* = 35; TNBC, *n* = 50).

### 4.5. 5-Aza-2′-deoxycytidine (5-Aza-dC) Treatment

1 × 10^5^ breast cancer cells were seeded into a 60 mm plate 12 h before treatment. Cells were treated with 5-Aza-2′-deoxycytidine (A3656, Sigma-Aldrich) at a concentration of 2 µM in the growth medium. The growth medium and 5-Aza-dC treatment were refreshed every 24 h for a total of 120 h.

### 4.6. Amplicon-Based Library Preparation and Targeted Bisulfite Sequencing

Genomic DNA (1 µg) was converted by sodium bisulfite treatment with EZ DNA Methylation Kit (Zymo Research, Irvine, CA, USA) according to the manufacturer’s instructions. A first amplification step was carried out on bisulfite DNA using the following MPPED2 specific primers: Forward (Fw) = 5′aaaTTaatTTaaagtagagaat 3′; Reverse (Rv) = 5′cttttatAcccacttccaAttac 3′. Capital letters are referred to the C or G after bisulfite treatment. At each primer overhang adaptor sequences (Fw: 5′-TCGTCGGCAGCGTCAGATGTGTATAAGAGACAG-3′; Rv: 5′-GTCTCGTGGGCTCGGAGATGTGTATAAGAGACAG-3′) were added. The PCR reaction was conducted according to the following conditions: denaturation at 95 °C for 2 min; 35 cycles of denaturation at 95 °C for 30 s, annealing at 52 °C for 40 s, and extension at 72 °C for 50 s. Final elongation at 72 °C was conducted for 6 min. A second PCR step was performed to add Illumina multiplexing indices (“Nextera XT” primers, Illumina, San Diego, CA, USA) that allow samples identification after sequencing. Two purification steps were performed using AMPure magnetic beads (Beckman-Coulter, Brea, CA, USA) following the manufacturer’s protocol. After amplicons quantification using Qubit^®^ 2.0 Fluorometer (Life Technologies), an equimolar library of bisulfite-treated amplicons was prepared and then diluted to final concentration of 8 picomolar. To increase diversity in base calling during sequencing, Phix control library was added [8% (*v*/*v*)]. Amplicons library was subjected to sequencing using V2 reagents kits on Illumina MiSeq system (Illumina). Paired-end sequencing was performed in 250 cycles per read (250 × 2). An average of 200,000 reads for sample were obtained. For the bioinformatics analyses, paired-end reads were assembled together with a minimum of 40 overlapping residues as threshold with the PEAR tool [30]. FASTQ assembled reads were then converted in FASTA format using the PRINSEQ tool [31]. To analyze the methylation status of each amplicon, we used AmpliMethProfiler pipeline software [32,33], a phyton-based pipeline specifically designed for deep-targeted bisulfite amplicon sequencing. AmpliMethProfiler produces quality filtered FASTA and directly extracts average methylation comparing each sequence with a gene-specific reference file in the FASTA format.

### 4.7. RNA Immunoprecipitation (RIP) Assay

RIP experiments were performed using the Magna RIP™ RNA-Binding Protein Immunoprecipitation Kit (Millipore, Billerica, MA, USA) according to the manufacturer’s protocol. Briefly, SKBR3 cells were harvested and lysed in complete RIP lysis buffer. 5 µg of human anti-DNMT1 antibody (#ab13537, Abcam, Cambridge, UK) and normal mouse IgG (Millipore), used as negative control, were incubated with magnetic beads for 30 min. Then, 100 μL of whole lysates were incubated overnight on a rocking platform at 4 °C. Next day, samples were incubated with Proteinase K buffer at 65 °C for 30 min and then immunoprecipitated RNA was purified. Purified RNA was reverse transcribed into cDNA by using random primer with the QuantiTect Reverse Transcription Kit (Qiagen, Hilden, Germany), and analyzed by qRT-PCR.

### 4.8. Immunohistochemical Evaluation of A Breast Tissue Microarray (TMA)

A human breast cancer TMA was purchased from Super Bio Chips (Super Bio Chips Laboratories, Seoul, Korea). The TMA section was deparaffinized in xylene (2× 10 min) and re-hydrated in ethanol solutions at decreasing concentration (from 100% to 50%). The TMA slide was then permeabilized in phosphate-buffered saline (PBS)-0.2% triton (5 min), washed 2× 5 min with PBS and, subsequently, it underwent unmasking treatment in citrate buffer (0.01 M pH6) for 15 min in microwave. Endogenous peroxidases were then blocked with methanol and 1.5% oxygen peroxide and tissues were once again permeabilized with PBS-0.2% triton for 5 min, washed 2× 5 min in PBS and blocked in blocking solution (5% normal goat serum, 3% BSA, 20 mM MgCl_2_, 0.3% tween 20 in PBS) for 1 h at RT. The rabbit polyclonal MPPED2 antibody (H00000744-D01P, Abnova, Taipei City, Taiwan) was used 1:50 in blocking solution overnight at 4 °C. The section then underwent the following protocol: PBS-0.2% triton for 5 min, PBS 2× 5 min, 1 h biotinylated α-rabbit IgG, H+L secondary antibody (BA-1000, Vector Laboratories, Burlingame, CA, USA) 1:100 in blocking solution for 1 h at RT, PBS-0.2% triton for 5 min, PBS 2× 5 min, ABC (SK-4000, Vector Laboratories) for 30 min at RT, PBS-0.2% triton for 5 min, PBS 3× 5 min, DAB substrate (SK-4100, Vector Laboratories). The slide was then de-hydrated and covered with glass using D. P. X. mountant liquid (GRM655, Sigma-Aldrich) and finally acquired with a NanoZoomer Digital Pathology System (Hamamatsu, Shizuoka, Japan). TMA comprised *n* = 59 total cases including, *n* = 40 primary tumor samples, *n* = 10 metastatic samples and *n* = 9 normal adjacent tissues. However, only *n* = 54 samples, including *n* = 38 primary tumors, *n* = 10 metastases, *n* = 6 normal adjacent tissues were evaluable. Two pathologists evaluated slide independently. For each tissue spot, intensity of the staining and percentage of positive cells were recorded. The intensity of the staining was scored from 0 to 3+, where 0 is no staining, 1+ is weak staining, 2+ is moderate staining and 3+ is strong staining. H-score was calculated according to the following formula: 1 × (% of 1+ cells) + 2 × (% of 2+ cells) + 3 × (% of 3+ cells), to assign to each sample an expression value based on the percentage of MPPED2 expressing cells and the intensity of staining [34,35].

### 4.9. RNA Extraction and Quantitative Real-Time Polymerase Chain Reaction (qRT-PCR)

Total RNA from breast tissues and cell lines was extracted using Trizol reagent (Life Technologies). 1 µg of total RNA from each sample was used to obtain double strand cDNA with the QuantiTect Reverse Transcription Kit (Qiagen). qRT-PCR was performed with the CFX96 thermocycler (Bio-Rad, Hercules, CA, USA) in 96-well plates. For each PCR reaction, 10 µL of 2× Sybr Green (Bio-Rad), 200 nM of each primer and 20 ng of the cDNA, previously generated, were used. The oligonucleotides for qRT-PCR, encompassing exon-exon junctions, were purchased from Integrated DNA Technologies (San Diego, CA, USA) and designed with Primer-BLAST software. Sequences are as follows:

- MPPED2 Fw: 5′-GCTTCAAAGAGTGGGCTGTG-3′, Rv: 5′-GAGGGTTGGTCGGTTGAAAG-3′
- RP18S Fw: 5′-TGCGAGTACTCAACACCAA-3′, Rv: 5′-TTGGTGAGGTCAATGTCTGC-3′
- MPPED2-AS1 Fw: 5′-TGGTGCAGGGATTGTTGCAT-3′, Rv: 5′-TGAACGACTGCAACTGCTTTG-3′.

Relative gene expression was determined using comparative C(t) method, as described elsewhere [36,37]. RP18S was used as housekeeping gene.

### 4.10. Western Blot

Cells were homogenized in RIPA lysis buffer (20 mM Tris-HCl pH 7.5, 5 mM Ethylenediaminetetraacetic acid (EDTA), 150 mM NaCl, 1% Nonidet P40, and a mix of protease inhibitors). Cell protein lysates were then subjected to sodium dodecyl sulfate polyacrylamide gel electrophoresis (SDS-PAGE) and transferred onto Immobilon-P transfer membranes (Merck Millipore, Burlington, MA, USA). Membranes were blocked with 5% non-fat milk and probed with the indicated antibodies at the appropriate dilutions: MPPED2 (1:500; NBP1-80499, Novus Biologicals, Centennial, CO, USA), cyclin D1 (1:1000; sc-718, Santa Cruz Biotechnology, Santa Cruz, CA, USA), cyclin E (1:1000; sc-248, Santa Cruz Biotechnology), ZEB1 (D80D3) (1:500, #3396S, Cell Signaling, Danvers, MA, USA), E-cadherin (24E10) (1:500; #3195, Cell Signaling), β-actin (1:5000; A5441, Sigma-Aldrich) and α-tubulin (1:10,000; T6074, Sigma-Aldrich). Membranes were incubated with horseradish peroxidase-conjugated secondary antibody (1:3000) for 60 min at RT. Signals were finally detected with chemiluminescent detection system (ECL) (Thermo Fisher Scientific, Waltham, MA, USA), and films were developed with a semiautomatic developing machine (Cawomat IR 2000, CAWO Photochemisches, Schrobenhausen, Germany). Densitometric analyses of the Western blot bands were evaluated by using ImageJ 1.43 software (NIH, Bethesda, MD, USA).

### 4.11. Flow Cytometry

Flow cytometry analyses were performed as previously described [38]. Briefly, breast cancer cells, seeded into a 100 mm plate, were trypsinized, washed twice in cold PBS and fixed with 70% ethanol, after 96 h. After centrifugation at 1200 rpm for 10 min at 4 °C, cells were treated with 50 µg/mL propidium iodide and 25 µg/mL RNase DNase-free (Roche, Basel, Switzerland) in PBS for 20 min at RT, safe of light. For each measurement 10,000 events were analyzed by employing a BD Accuri^TM^ C6 flow cytometer (BD Biosciences, Franklin Lakes, NJ, USA) and cell cycle data were analyzed with the BD Accuri C6 Software in a semiautomatic analysis procedure.

### 4.12. Cell Migration and Invasion Assays

Migration and invasion assays were performed using transwell chamber (8 μm pores). Invasion was estimated by using Matrigel (BD Biosciences). Briefly, human breast cancer cells (3 × 10^4^ for migration and 1 × 10^5^ for invasion) were plated in the upper transwell chamber in serum-free medium. Then, 0.3 mL complete medium were added in the lower chamber as chemoattractant. After 24 h (migration) and 48 h (invasion) of incubation, migrated or invaded cells on the membrane of the chambers were fixed and stained with crystal violet solution (crystal violet 0.05%, methanol 20%). After acquisition of images, crystal violet in the chamber was de-stained with PBS-0.1% SDS solution and was read at 590 nm in a microplate reader (LX800, Universal Microplate Reader, BioTek Instruments, Inc., Winooski, VT, USA). Breast cancer cell lines were also seeded in a 96 well plate to normalize the number of used cells. After two hours, the absorbance at 490 nm was read using cell titer (Promega, Fitchburg, WI, USA). Results were obtained by normalizing the crystal violet values to cell titer ones [13].

### 4.13. Colony Formation Assay

MDA-MB-231 and SKBR3 cells at 80% of confluency in 100 mm plate were transfected with pCDNA3.1-EV and pCDNA3.1-MPPED2. 24 h after transfection, cells were treated with 600 µg/mL and 100 µg/mL hygromycin, respectively. Medium containing hygromycin was refreshed every two days and, after 3 weeks of hygromycin selection, cells were fixed and stained with a solution containing crystal violet.

### 4.14. Growth Curve Assay

For the growth curve assay, 3 × 10^4^ breast cancer cells were plated in a 60 mm plate. Cells were counted in triplicate with Burker hemocytometer chamber to evaluate cell growth rate for 5 days.

### 4.15. Statistical Analysis

All results were reported as mean ± standard deviation (SD). Data were analyzed by Student’s *t*-test, Mann–Whitney’s test and ANOVA test, when required. The correlations were evaluated through non-parametric Spearman’s rank correlation coefficient with 95% confidence interval. To assess the relationship between protein expression levels and clinico-pathological features, Fisher’s exact test was used. Statistical analyses were carried out using GraphPad Prism software 6.0 and the difference was considered significant when *p* < 0.05.

## 5. Conclusions

In conclusion, the results presented here support a tumor suppressor role of MPPED2 in breast carcinogenesis and propose the hypermethylation of its promoter as one of the main mechanisms accounting for its downregulation in BC. Intriguingly, reduced expression levels of *MPPED2-AS1* lncRNA let to increase the activity of DNMT1, which in turn is responsible of the hypermethylation of *MPPED2* promoter.

## Figures and Tables

**Table 1:** MPPED2 protein expression evaluated by immunohistochemistry in breast cancer (BC)

Histological Type	MPPED2 Staining Score ^a^, *n* (%)			
	0	1+	2+	3+
Normal adjacent tissues (*n* = 6)	0 (0)	0 (0)	0 (0)	6 (100)
Primary BC tissue (*n* = 38)	3 (7.8)	10 (26.3)	12 (31.5)	13 (34.2)
BC metastatic carcinoma (*n* = 10)	0 (0)	3 (30)	4 (40)	3 (30)

^a^ 0, no signal; 1+, weak signal; 2+, moderate signal; 3+, strong signal.

## Supplementary Materials

The following are available online at https://www.mdpi.com/2072-6694/11/6/797/s1: Figure S1: Analysis of *MPPED2* and *MPPED2-AS1* expression in the TCGA dataset. (A) *MPPED2-AS1* expression levels were evaluated in The Cancer Genome Atlas (TCGA) dataset. Paired (left panel) and non-paired (right panel) breast cancer samples were evaluated. *t*-test: ****, *p* < 0.0001 (primary tumors vs. normal tissues in both paired and non-paired samples). (B) *MPPED2-AS1* expression levels were evaluated in the molecular subtypes (Lum A, *n* = 23; Lum B, *n* = 6; HER2, *n* = 6; TNBC, *n* = 9) of the TCGA dataset. No statistical significance was observed among the groups (One-way ANOVA, *p* = 0.29). (C) *MPPED2* expression levels were evaluated in the TCGA dataset. ER+ (*n* = 172), ER− (*n* = 88) and normal (*n* = 61) breast samples were evaluated. One-way ANOVA: ****, *p* < 0.0001 (ER+ vs. normal and ER− vs. normal); ***, *p* < 0.001 (ER+ vs. ER−), Figure S2: Expression analysis of *MPPED2* in human breast carcinoma cell lines. qRT-PCR analysis of *MPPED2* mRNA levels in a panel of human breast carcinoma cell lines, including MDA-MB-231, SKBR3, MDA-MB-468, BT-474 and MCF7. Data are reported as 2^−ΔCt^ values ± SD, Figure S3: *MPPED2* reduces proliferation and migration of breast carcinoma cell lines. (A,B) qRT-PCR performed in MDA-MB-468 and MCF7 cell lines transiently transfected with *MPPED2* or carrying the corresponding empty vector (EV) (left panel). Data are reported as 2^−ΔΔCt^ values ± SD, compared to the EV, set equal to 1. Western bot analysis confirming the expression of MPPED2 (right panel). β-actin was used to normalize the amount of loaded protein. Densitometric analysis was performed by using ImageJ software to evaluate MPPED2 protein overexpression compared to EV, set equal to 1, after normalization. Original blots were shown in Figure S8. (C) Cell growth analysis was performed in MDA-MB-468 (left panel) and MCF7 (right panel) cells transiently expressing *MPPED2* or carrying the corresponding empty vector (EV). Cell number was evaluated at 24 h, 48 h and 72 h after seeding. Values were obtained from three independent experiments. Data were reported as mean ± SD. 2-way ANOVA test (Bonferroni post-test: *MPPED2* vs. EV, 72 h, ***, *p* < 0.001) (D) Representative images of migration assays performed in MDA-MB-468 and MCF7 cells transiently transfected with *MPPED2* or the corresponding empty vector (EV). Magnification 40×, Figure S4: Original blots for Western blot analyses to Figure 3: (A,B) Western blot images of Figure 3F relative to MPPED2 and α-tubulin in MDA-MB-231, respectively. (C,D) Western blot images of Figure 3G relative to MPPED2 and to α-tubulin in SKBR3, respectively. Figure S5: Original blots for Western blot analyses to Figure 5: (A,B) Western blot images of Figure 5A relative to MPPED2 and β-actin in MDA-MB-231, respectively, (C,D) Western blot images of Figure 5B relative to MPPED2 and β-actin in SKBR3, respectively. Figure S6:Original blots for Western blot analyses to Figure 6: (A,B,C) Western blot images of Figure 6C relative to β-actin, Cyclin D and Cyclin E in SKBR3, respectively, (D,E,F) Western blot images of Figure 6C relative to β-actin, Cyclin D and Cyclin E in MDA-MB-231, respectively. Figure S7: Original blots for Western blot analyses to Figure 7: (A) Western blot image of Figure 7C relative to β-actin in SKBR3 (upper panel), Western blot image of Figure 7C relative to β-actin in MDA-MB-231 and SKBR3 cells (lower panel), (B) Western blot image of Figure 7C relative to E-cadherin in SKBR3 (upper panel), Western blot image of Figure 7C relative to ZEB1 in MDA-MB-231 and SKBR3 cells (lower panel), (C,D) Western blot images of Figure 7C relative to E-cadherin and β-actin in MDA-MB-231, respectively. Figure S8: Original blots for Western blot analyses to Figure S3: (A,B) Western blot image of Figure S3A and Figure S3B relative to β-actin and MPPED2 in MCF7 and MDA-MB-468 cells, respectively. Table S1: Association of MPPED2 expression and TMA breast cancer characteristic.
