# Supplementary material for: The Metallophosphoesterase-Domain-Containing Protein 2 (MPPED2) Gene Acts as Tumor Suppressor in Breast Cancer
Source: Cancers (Basel). 2019 Jun 8;11(6):797. doi: 10.3390/cancers11060797 (PMC6627064; doi:10.3390/cancers11060797)
Supplement: Supplementary file 1 [file cancers-11-00797-s001.pdf]

cancers

MDPI

Supplementary Materials: The *Metallophosphoesterase-Domain-Containing Protein 2* (*MPPED2*) Gene Acts as Tumor Suppressor in Breast Cancer

Simona Pellecchia, Romina Sepe, Antonella Federico, Mariella Cuomo, Sara Carmela Credendino, Pasquale Pisapia, Claudio Bellevicine, Pedro Nicolau-Neto, Mariana Severo, Elvira Crescenzi, Gabriella De Vita, Luigi Maria Terracciano, Lorenzo Chiariotti, Alfredo Fusco and Pierlorenzo Pallante

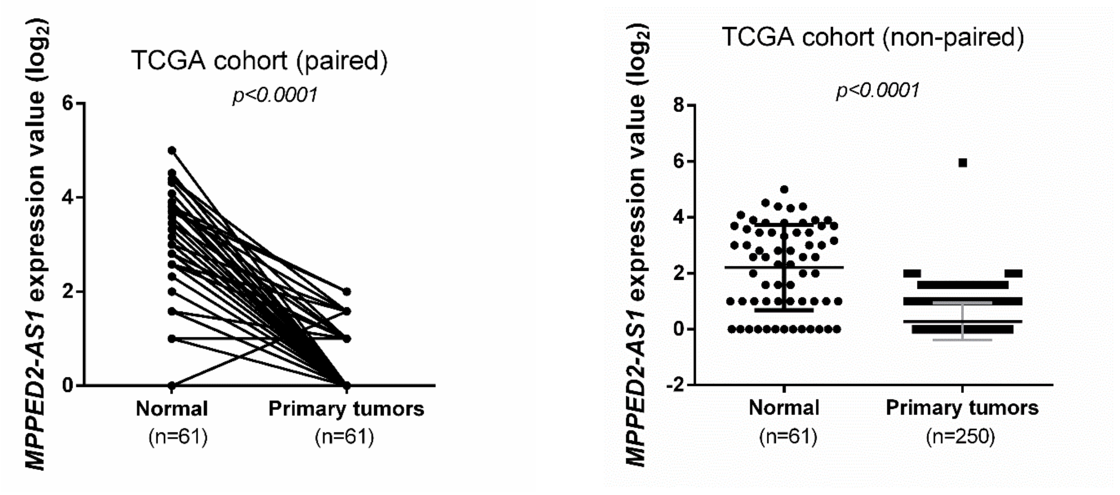

(**A**)

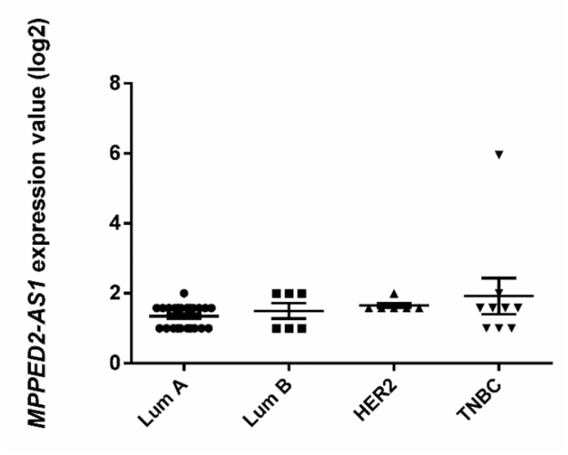

(**B**)

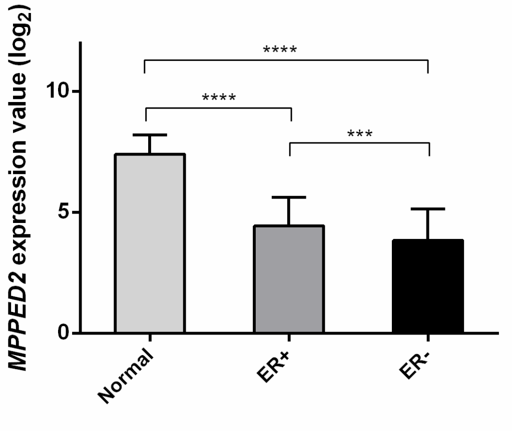

(**C**)

Figure S1. Analysis of *MPPED2* and *MPPED2-AS1* expression in The Cancer Genome Atlas (TCGA) dataset. (**A**) *MPPED2-AS1* expression levels were evaluated in TCGA dataset. Paired (left panel) and non-paired (right panel) breast cancer samples were evaluated. *t*-test: ****, $p < 0.0001$ (primary tumors vs. normal tissues in both paired and non-paired samples). (**B**) *MPPED2-AS1* expression levels were evaluated in the molecular subtypes (Lum A, $n = 23$; Lum B, $n = 6$; HER2, $n = 6$; TNBC, $n = 9$) of the TCGA dataset. No statistical significance was observed among the groups (one-way analysis of variance (ANOVA), $p = 0.29$). (**C**) *MPPED2* expression levels were evaluated in TCGA dataset. ER+ (*n*

= 172), ER− (n = 88) and normal (n = 61) breast samples were evaluated. One-way ANOVA: ****, $p <$ 0.0001 (ER+ vs. normal and ER− vs. normal); ***, $p < 0.001$ (ER+ vs. ER−).

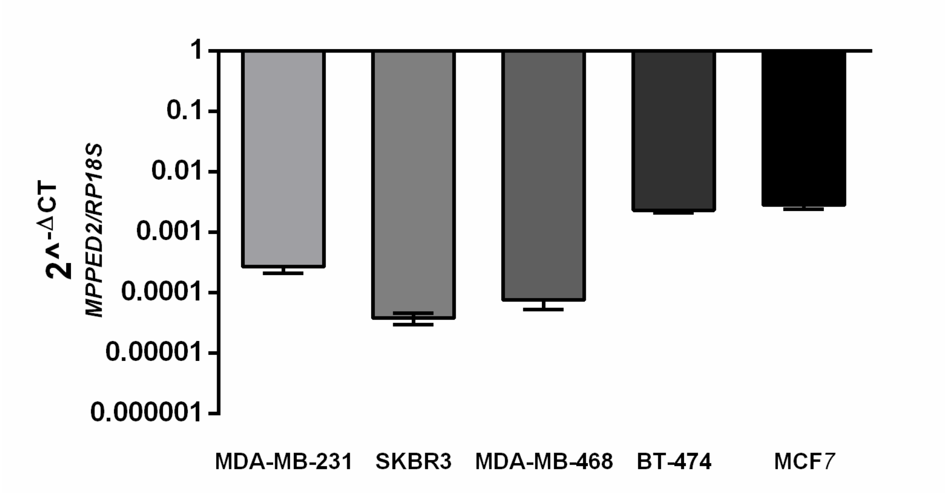

Figure 2. Expression analysis of *MPPED2* in human breast carcinoma cell lines. quantitative real-time polymerase chain reaction (qRT-PCR) analysis of *MPPED2* mRNA levels in a panel of human breast carcinoma cell lines, including MDA-MB-231, SKBR3, MDA-MB-468, BT-474 and MCF7. Data are reported as $2^{-\Delta Ct}$ values ±SD.

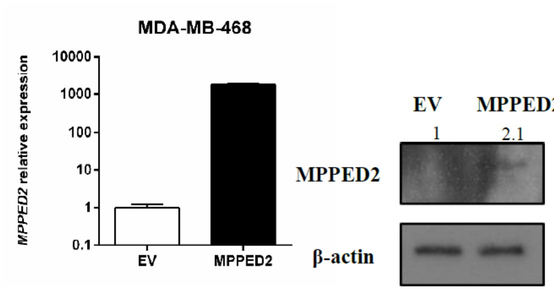

(**A**)

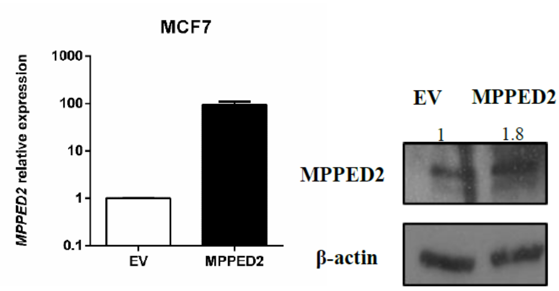

(**B**)

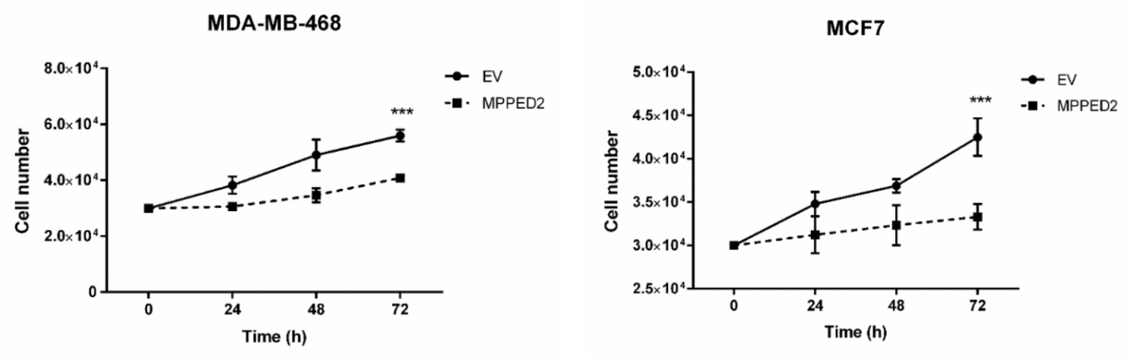

(**C**)

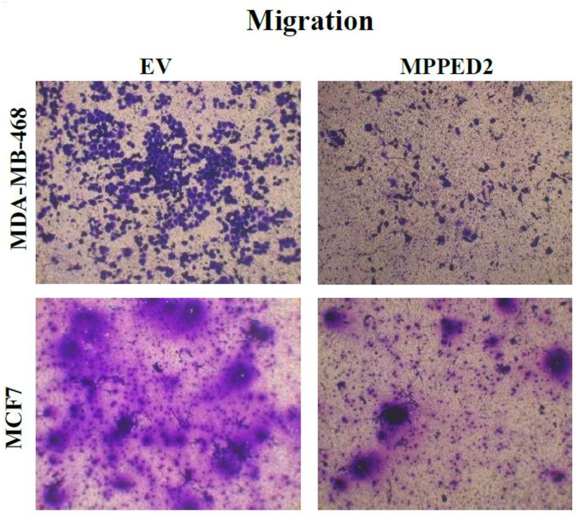

(**D**)

Figure S3. *MPPED2* reduces proliferation and migration of breast carcinoma cell lines. (**A**,**B**) qRT-PCR performed in MDA-MB-468 and MCF7 cell lines transiently transfected with *MPPED2* or carrying the corresponding empty vector (EV) (left panel). Data are reported as $2^{-\Delta\Delta Ct}$ values ±SD, compared to the EV, set equal to 1. Western blot analysis confirming the expression of MPPED2 (right panel). β-actin was used to normalize the amount of loaded protein. Densitometric analysis was performed by using ImageJ software to evaluate MPPED2 overexpression compared to EV, set equal to 1. (**C**) Cell growth analysis was performed in MDA-MB-468 (left panel) and MCF7 (right panel) cells transiently expressing MPPED2 or carrying the corresponding empty vector (EV). Cell number was evaluated at 24 h, 48 h and 72 h after seeding. Values were obtained from three independent experiments. Data were reported as mean ±SD. Two-way ANOVA test (Bonferroni post-test: MPPED2 vs. EV. 72h, ***, $p < 0.001$) (**D**) Representative images of migration assays performed in MDA-MB-468 and MCF7 cells transiently transfected with *MPPED2* or the corresponding empty vector (EV). Magnification 40×.

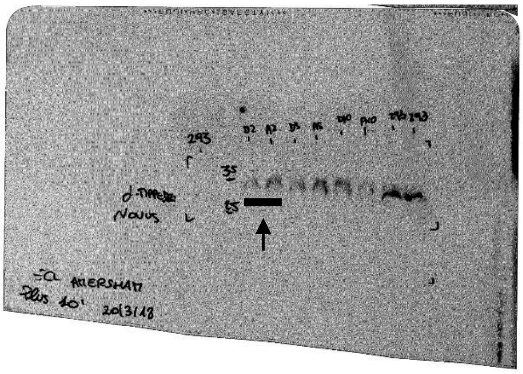

(**A**)

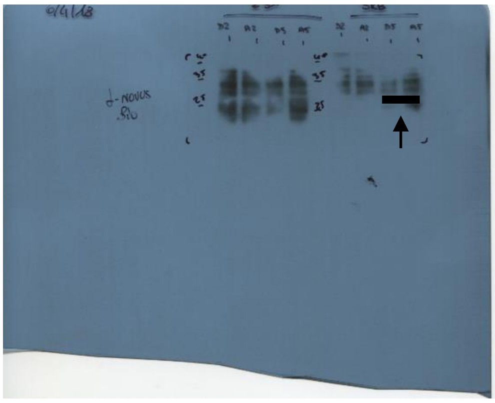

(**B**)

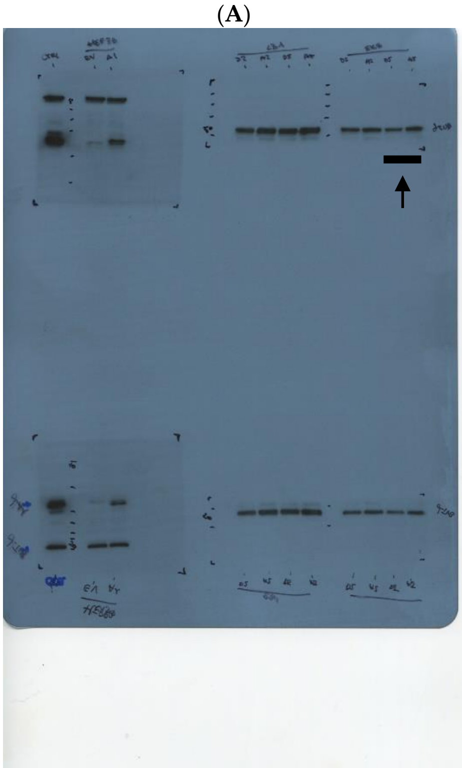

(**C**)

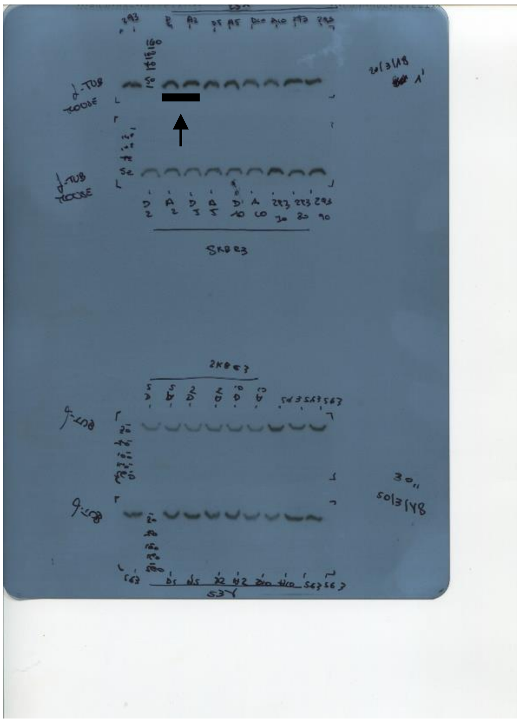

(**D**)

Figure S4. Supplementary Materials for Western blot to Figure 3: (**A**) Figure 3F MPPED2, (**B**) Figure 3G MPPED2, (**C**) Figure 3G α-tubulin (**D**) Figure 3F α-tubulin.

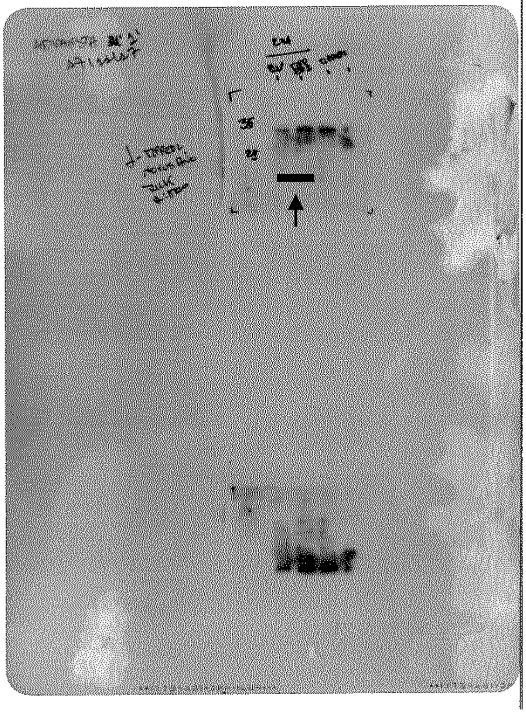

(**A**)

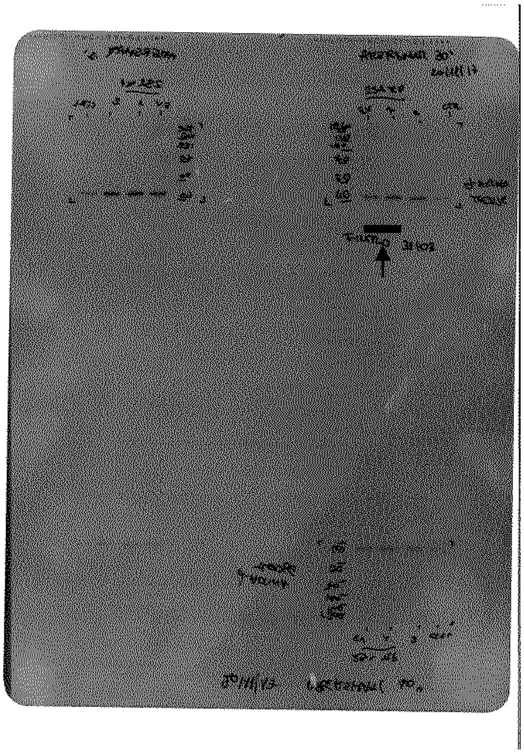

(**B**)

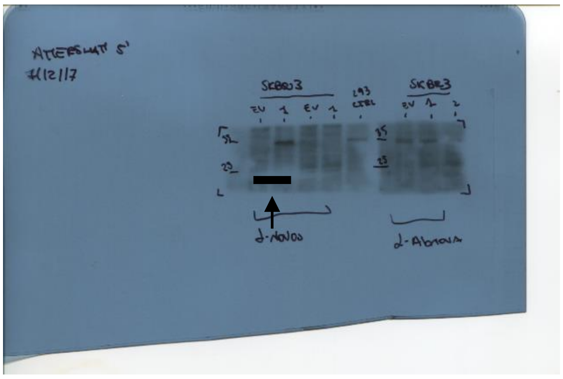

(**C**)

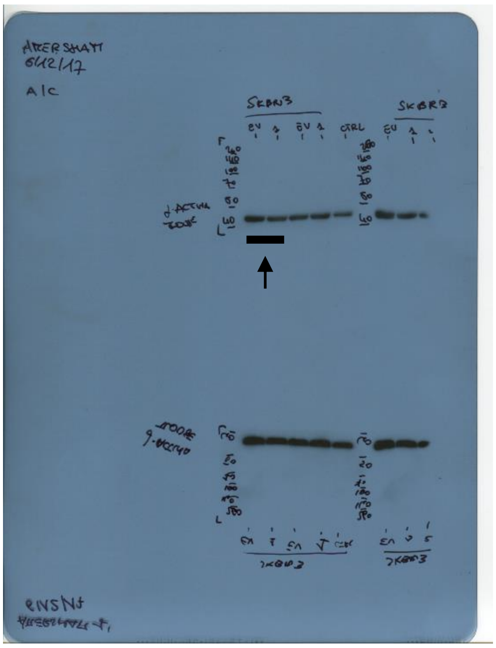

(**D**)

Figure S5. Supplementary Materials for Western blot to Figure 5: (**A**) Figure 5A MPPED2, (**B**) Figure 5A β-actin, (**C**) Figure 5B MPPED2, (**D**) Figure 5B β-actin.

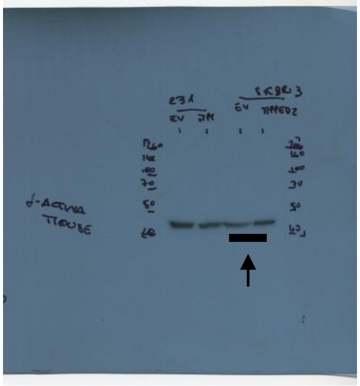

(**A**)

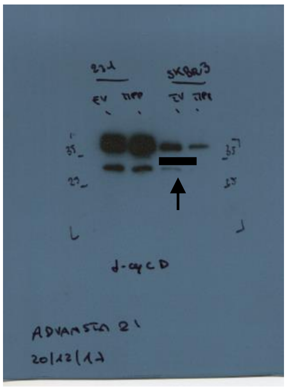

(**B**)

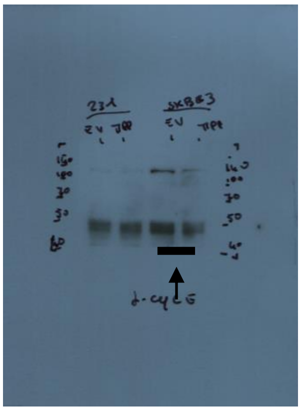

(**C**)

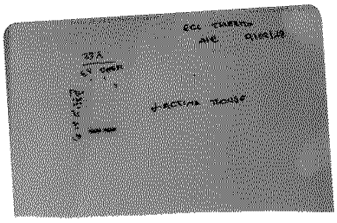

(**D**)

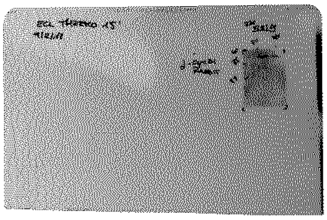

(**E**)

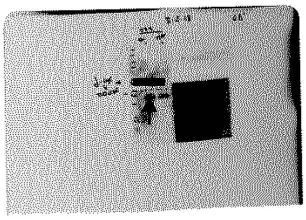

(**F**)

Figure S6. Supplementary Materials for Western blot to Figure 6: (**A**) Figure 6C SKBR3 β-actin, (**B**) Figure 6C SKBR3 Cyclin D, (**C**) Figure 6C Cyclin E, (**D**) Figure 6C MDA-MB-231 β-actin, (**E**) Figure 6C MDA-MB-231 Cyclin D, (**F**) Figure 6C MDA-MB-231 Cyclin E.

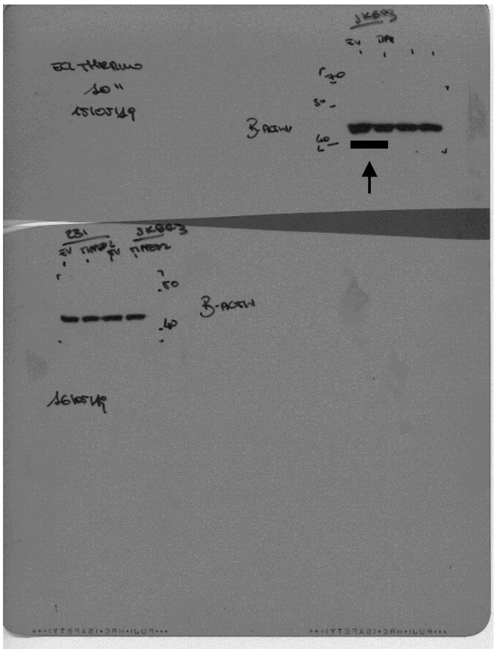

(**A**)

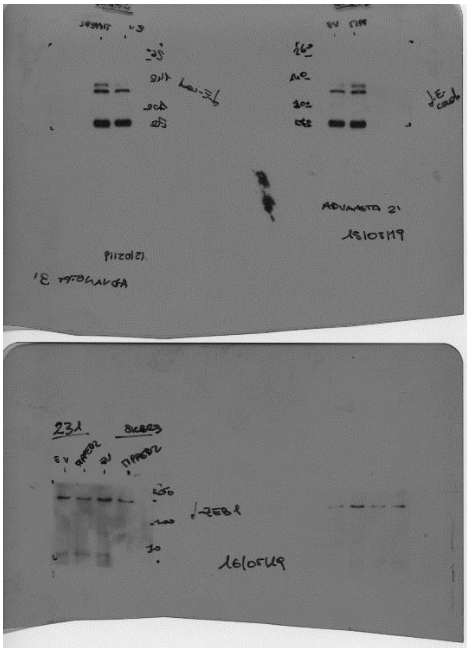

(**B**)

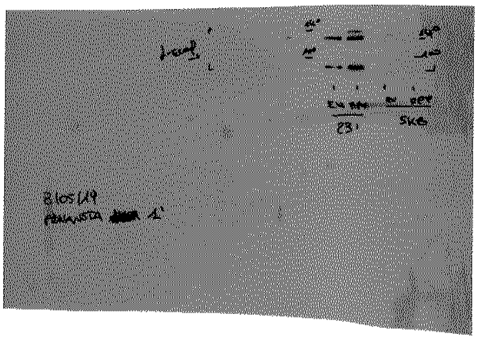

(**C**)

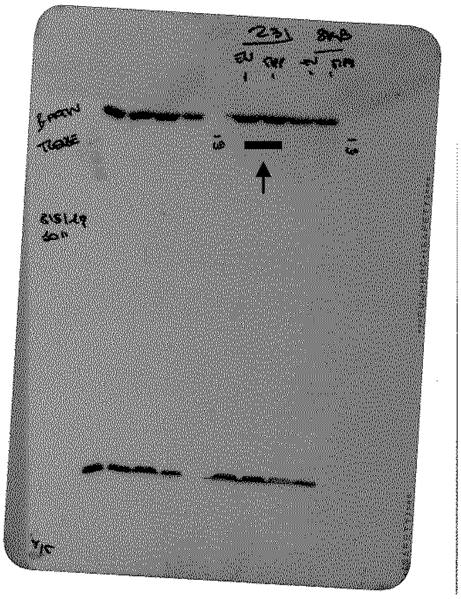

(**D**)

Figure S7. Supplementary Materials for Western blot to Figure 7: (**A**) Figure 7C SKBR3, β-actin, MDA-MB-231-SKBR3, β-actin, (**B**) Figure 7C SKBR3, E-cadherin, MDA-MB-231, SKBR3, ZEB1, (**C**) Figure 7C MDA-MB231, E-cadherin, (**D**) Figure 7C MDA-MB-231, β-actin.

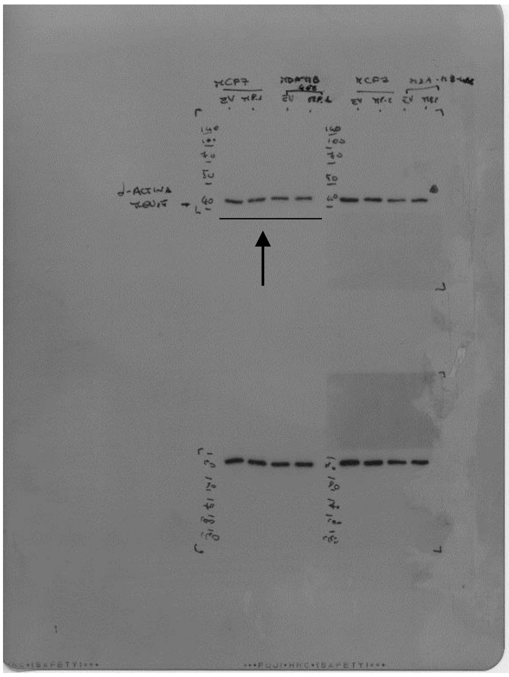

(**A**)

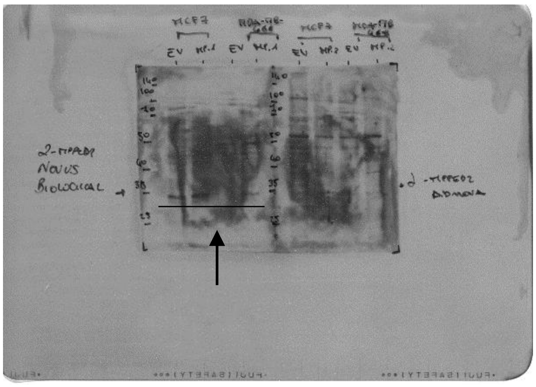

(**B**)

Figure S8. Supplementary Materials for Western blot to Figure S3: (**A**) Figure S3A and S3B, β-actin, (**B**) Figure S3A and S3B, MPPED2.

Table S1. Association of MPPED2 expression and tissue microarray (TMA) breast cancer characteristic.

**Characteristic**	***n***	**MPPED2 Staining**		***p*-Value***
		**Low (0,1+) *n* (%)**	**High (2+,3+) *n* (%)**	
Age				
<50	22	12 (31.6)	10 (26.3)	0.0002
≥50	16	1 (2.6)	15 (39.5)	
Tumor size				
≤3 cm	16	5 (13.2)	12 (31.6)	0.734
>3 cm	22	8 (21)	13 (34.2)	
Nottingham histological grade				
I	0			0.968
II	16	6 (15.8)	10 (26.3)	
III	19	7 (18.4)	12 (31.6)	
NA	3		3 (7.9)	
Lymph node				
Negative	13	3 (7.9)	10 (26.3)	0.473
Positive	25	10 (26.3)	15 (39.5)	
Estrogen receptor				
Negative	27	11 (28.9)	16 (42.1)	0.267
Positive	11	2 (5.3)	9 (23.7)	
Progesterone receptor				
Negative	28	11 (29)	17 (44.7)	0.441
Positive	10	2 (5.3)	8 (21)	
Her2				
Negative	28	9 (23.7)	19 (50)	0.709
Positive	10	4 (10.5)	6 (15.8)	
Tumor stage				
I	0			0.295
II	23	6 (15.8)	17 (44.7)	
III	15	7 (18.4)	8 (21)	
**Total**	**38**			

* Fisher's exact test: samples were grouped in low (0, 1+) and high (2+, 3+) expressors, based on the intensity of the staining. NA, not available.

© 2019 by the authors. Submitted for possible open access publication under the terms and conditions of the Creative Commons Attribution (CC BY) license (http://creativecommons.org/licenses/by/4.0/).
